# Vacuum-assisted core biopsy of the breast: a 3-year single-centre experience

**DOI:** 10.1186/bcr3315

**Published:** 2012-11-09

**Authors:** M Al-Attar, MM Hoosein, K Wren, D Lister, E Denton, G McDonald, S Kaneri, L Grosvenor

**Affiliations:** 1 University Hospitals of Leicester, UK

## Introduction

Vacuum-assisted core biopsy (VACB) of the breast is a minimally invasive technique, used increasingly for the assessment of mammographically and ultrasound detected, nonpalpable breast lesions. The effectiveness of VACB has been demonstrated on lesions both with and without microcalcifications. VACB allows the operator to obtain a sufficient specimen with a single insertion to provide for a more accurate diagnosis.

## Methods

The aim of this study was to audit the use and effectiveness of ultrasound and stereotaxis guided VACB at a single centre over a 3-year period. We retrospectively identified patients from the Leicestershire Breast Screening Service and Radiology Information Systems records. A total of 152 patients undergoing 157 VACB (*n *= 157) were identified. Of these, 133 were stereotaxis guided, the remaining 24 ultrasound guided. The overall lesion workload was as follows: asymmetrical densities = 6 (4%); mass = 31(20%); parenchymal distortion = 32(20%); and calcification = 88(56%).

## Results

A positive histological diagnosis was achieved in 153 (97%) of the total VACBs performed. Of these patients, 136 (88.9%) had a prior 14G core biopsy, 16 (10.5%) had VACB as a first-line procedure. Outcomes for VACB were then compared against 14G biopsies for individual histological grade, presented as follows - grade on 14G needle (definitive diagnosis percentage on VACB): B1 (83%); B2 (67%); B3 (62%); B4 (76%); and B5c (100%). Overall, 51 (33.6%) patients avoided surgery for benign breast disease (95% CI = 26.1 to 41.1%), and 33 patients (21.7%) had single definitive surgery thereby avoiding multiple procedures (95% CI = 15.2 to 28.3%).

## Conclusion

VACB is a safe, well-tolerated and extremely useful sampling modality which can, in selective cases, accurately determine onward surgical management and avoid unnecessary surgery for benign breast disease.

